# The effects of an Internet based self-help course for reducing panic symptoms - Don't Panic Online: study protocol for a randomised controlled trial

**DOI:** 10.1186/1745-6215-12-75

**Published:** 2011-03-11

**Authors:** Wouter van Ballegooijen, Heleen Riper, Annemieke van Straten, Jeannet Kramer, Barbara Conijn, Pim Cuijpers

**Affiliations:** 1 Department of clinical psychology and EMGO Institute, VU-University, Amsterdam, The Netherlands; 2 Trimbos Institute (Netherlands Institute of Mental Health and Addiction), Utrecht, The Netherlands; 3 GGZ InGeest, Amsterdam, The Netherlands

## Abstract

**Background:**

Internet based self-help for panic disorder (PD) has proven to be effective. However, studies so far have focussed on treating a full-blown disorder. Panic symptoms that do not meet DSM-IV criteria are more prevalent than the full-blown disorder and patients with sub-clinical panic symptoms are at risk of developing PD. This study is a randomised controlled trial aimed to evaluate an Internet based self-help intervention for sub-clinical and mild PD compared to a waiting list control group.

**Methods:**

Participants with mild or sub-clinical PD (N = 128) will be recruited in the general population. Severity of panic and anxiety symptoms are the primary outcome measures. Secondary outcomes include depressive symptoms, quality of life, loss of production and health care consumption. Assessments will take place on the Internet at baseline and three months after baseline.

**Discussion:**

Results will indicate the effectiveness of Internet based self-help for sub-clinical and mild PD. Strengths of this design are the external validity and the fact that it is almost completely conducted online.

**Trial registration:**

Netherlands Trial Register (NTR): NTR1639 The Netherlands Trial Register is part of the Dutch Cochrane Centre.

## Background

Anxiety disorders are highly prevalent, affecting one in six people during their lifetime [1], and cause a substantial loss of quality of life [2]. Direct and indirect costs caused by anxiety disorders are estimated at half a billion dollars per 1 million adults per year [3]. One of those anxiety disorders is panic disorder (PD). A study among the population in the Netherlands shows PD affects 1.5% of all adults each year, while 2% of the population is affected by sub-clinical PD [4]. Sub-clinical PD indicates infrequent panic attacks or frequent panic attacks that are relatively mild. It can be defined as panic symptoms that do not meet DSM-IV criteria for PD. Sub-clinical PD is a substantial burden for both the patient and society [4]. Panic symptoms are often comorbid with other mental health problems, like depression or other anxiety disorders [5]. Comorbid anxiety and depressive symptoms can lead to suicidal ideation [6] and PD elevates the risk at suicide attempts when comorbid with a mood disorder, substance abuse or another anxiety disorder [7].

While PD can be effectively treated by cognitive behavioural therapy and pharmacotherapy [8], it is known that only 25% of people with anxiety symptoms seeks help [9] and only 15% of people with panic symptoms receives effective treatment [10]. Help-seeking might be too difficult or fearful for people with panic symptoms, especially when they suffer from agoraphobia as well. In addition, help-seeking could be further compromised by fear of stigmatisation, misinterpretation of panic symptoms as a physical problem, or little awareness of effective treatment opportunities or available services.

A more accessible and perhaps more acceptable alternative to face-to-face interventions for PD is Internet based self-help. Several studies show promising effects of Internet based self-help for PD [11-13]. Moreover, Internet based self-help courses save clinicians' time and cost little compared to face-to-face treatment or pharmacotherapy [14].

Internet based self-help interventions for panic symptoms focus mainly on full-blown PD [11-13]. An accessible self-help course, tailored for the large group of sub-clinical and mild cases, would be beneficial to a group of patients that is not yet targeted by Internet interventions. Moreover, an Internet based self-help course for sub-clinical and mild panic symptoms could close the gap between prevention and early intervention of panic disorder.

This study is designed to evaluate Don't Panic Online (*Geen Paniek Online*) [15], an Internet based self-help course for sub-clinical and mild cases of PD with minimal guidance. Don't Panic Online (DPO) is based on Don't Panic (*Geen Paniek*) [16], a face-to-face group course for sub-clinical and mild panic symptoms. Don't Panic has proven to be cost-effective [17,18].

We will conduct a randomised controlled trial, comparing an experimental group that takes the course Don't Panic Online to a waiting list control group that will get access to an information website. The research question is the following: what is the effect of the Internet based self-help course Don't Panic Online on sub-clinical to mild panic symptoms?

## Methods

### Design

We will conduct a randomised controlled trial with two arms: (a) Internet based self-help with minimal guidance, (b) waiting list control group with information website. The Medical and Ethical Committee of VU Medical Centre approved the study protocol.

### Study population

The study population consists of adults with sub-clinical or mild PD. The inclusion criteria are the following: 18 years of age or older, Internet access, sub-clinical or mild PD (PDSS-SR score 5-15). Consequently, individuals with too mild (PDSS-SR score 1-4) or too severe (PDSS-SR score 15 or higher) panic symptoms will be excluded. Participants who report moderate to high suicide risk, as measured by a self-report version of the MINI, will be excluded as well. Applicants who report severe panic symptoms or suicide risk will be contacted by e-mail and advised to contact their general practitioner.

### Sample size

Power calculations are based on a moderate effect size of d=.50, comparing the PDSS-SR scores of the intervention group to the control group with a two-sided t-test (alpha .05, power 80%). To show this effect each group will consist of 64 participants [19], so the total sample size adds up to N = 128. Missing values will be imputed using regression imputation.

### Recruitment

Participants will be recruited in the general population by means of a Facebook advertisement campaign, messages on panic or anxiety related online forums, banners on health related websites and advertisements in newspapers. Interested individuals will be directed to a study website, where they can find further information as well as an informed consent form. They can apply for participation by printing and signing the informed consent form, which can be scanned and sent by e-mail or sent by postal mail. After application, the researchers will send the participants a link to an online questionnaire.

### Randomisation and procedure

After screening and completion of the baseline questionnaires (t0), the participant will be contacted for a diagnostic interview by telephone. The interview will be conducted within two weeks after baseline by a trained and experienced interviewer. All participants will be randomised to one of the two groups, regardless of the presence or absence of a diagnosis. Randomisation will be stratified for the presence or absence of agoraphobia symptoms (PDSS-SR item 4 score > 1) and the use of medication. Randomisation lists are generated with a computer program. Blinding of the participants and researchers is not possible due to the design of this research. The post-treatment assessment (t1) is scheduled at 3 months after baseline. Both the baseline and the post-treatment assessments are self-report and will be conducted through the Internet.

### Intervention

Don't Panic Online (DPO) is an individual guided web-based self-help course, based on cognitive behavioural therapy. It was developed by the Trimbos Institute, which is the Netherlands Institute for Mental health and Addiction, in collaboration with GGNet, a Dutch mental health care institute. The course consists of six sessions, in which the participants will learn to control their panic symptoms by applying various cognitive behavioural techniques and skills. The course's format is based on Colour Your Life, an evidence based Internet intervention for depressive symptoms [20,21].

DPO is a protocolised treatment and consists of the following components: keeping a log of panic attacks; analysis of fearful situations; challenging thoughts that enable feelings of panic; replacing these thoughts by more realistic, constructive thoughts that reduce anxiety; behavioural exercises; and ranking exercises from manageable to difficult and carrying them out in order of difficulty. Each session consists of text, voice over, animated diagrams and video. A typical session will take about thirty minutes and consists of an introduction, a discussion of the previous lesson's homework, new theory and homework for the next week. The course is designed to be followed on a weekly basis until session five, while the sixth lesson can be followed four weeks after the fifth. The course can be completed in eight weeks.

Besides the lessons, the participant has several online resources at his or her disposal: a homework station, a panic attack log, a library for extra information, reading tips and a discussion board.

The DPO website contains an e-mail system for contact between a participant and a coach. For the current study, the coach will reply to questions about the course, its exercises and the participant's mental health. He will also contact the user weekly to ask after his progress. Participants will be supported for a maximum of three months. The coaches will consist of students who are in the final phase of their study of clinical psychology. All will receive a brief training.

Participants in the control group will receive access to DPO after completing the t1 measurement. In the mean time, they will have access to an information website about PD.

### Instruments

All variables will be measured at t0 and t1, except for demographic data, the diagnosis and satisfaction with the intervention. Demographic data and diagnosis are only measured at t0, while satisfaction with the intervention will only be measured at t1.

#### Demographic data

This section contains questions about age, gender and education.

#### Diagnosis

The Composite International Diagnostic Interview (CIDI) [22] will be used to ascertain the presence or absence of PD, other anxiety disorders and depression. The CIDI has been developed by the WHO and is an extensive, fully structured diagnostic interview to assess DSM-IV Axis-I diagnoses [22]. Only the subscales depression, PD, agoraphobia, GAD, social phobia and post-traumatic stress disorder will be used in this study. The reliability of these subscales is sufficient (inter-rater κ = .94 - .99; test-retest κ = .62 - .84) [23]. In this study, a trained interviewer will administer the CIDI by telephone.

#### Anxiety and panic symptoms

For severity of panic symptoms the Panic Disorder Severity Scale - Self Report (PDSS-SR) will be used. The PDSS is originally a face-to-face interview [24] and was adapted to self-report by Houck et al. [25]. The instrument contains seven items that assess the severity of seven dimensions of panic disorder and associated symptoms. The PDSS-SR generates a total score ranging from 0 to 28, with a higher score indicating more severe panic symptoms. The questionnaire has good psychometric properties with Cronbach's alpha = 0.92 and intraclass correlation coefficient = 0.81 [25]. In the current study, a score of 0-4 will count as no clinically relevant symptoms and 16 or higher as severe PD.

Anxiety symptoms in general will be measured with the Beck Anxiety Inventory (BAI) [26]. The BAI contains 21 short questions. Internal consistency is high, with Cronbach's alpha ranging from .90 to .94 [26]. The score varies from 0 to 63. A score of 30 or higher is interpreted as severe anxiety symptoms.

#### Depressive symptoms

Depressive symptoms we will be measured with the CES-D [27]. The CES-D is a 20-item self-report questionnaire. Every item ranges from 0 to 3 and the total score ranges from 0 (no feelings of depression) to 60 (severe feelings of depression). Radloff [27] reports high internal consistencies among different populations (.79 to .92). The optimal cut-off score varies between 16 and 27 [28]. In the current study, we will regard 27 and higher as a high score.

#### Suicidal risk

Suicidal risk and ideation will be measured with the specific section of the Mini-International Neuropsychiatric Interview (MINI) [29,30]. The MINI suicide section consists of 6 items and classifies subjects into four groups: no suicidal risk, low suicidal risk, moderate suicidal risk, and high suicidal risk. In this study, the items are self-report. Participants with moderate to high suicidal risk will be excluded from this study.

#### Quality of life

Quality of life will be measured with the Dutch version of the EuroQol Questionnaire (EQ-5D) [31,32]. This short list contains five dimensions: mobility, self-care, usual activities, pain/discomfort and anxiety/depression. The EQ-5 D generates a total of 243 unique health states, each of which is associated with a utility score ranging from 0 (poor health) to 1 (perfect health). The EQ-5 D is a validated instrument for measuring general health-related quality of life [31-33].

#### Loss of production and health care consumption

An indication of loss of production and use of care will be made with the Trimbos and Institute of Medical Technology Assessment Questionnaire on Costs Associated with Psychiatric Illness (TiC-P) [34]. This list consists of three parts. Part I measures health care consumption of individuals who suffer from mental disorders. Part II examines loss of production (indirect costs) and part III general, demographic variables. In the current study, part III will be omitted, because demographic variables are already measured elsewhere.

#### Satisfaction and track-and-trace

A track-and-trace system will keep a record of the dates participants of the intervention group log on or finish a lesson. This system will also pose a question after each lesson: "Was this lesson useful to you?", which can be answered on a 5-point Likert scale. At t1, these participants will receive questions about their experience with the intervention. This includes the number of lessons followed, the amount of time spent on homework, satisfaction with the intervention and satisfaction with the coaching.

### Analyses

Differences in demographic and baseline clinical characteristics will be computed with Chi-square tests, *t*-tests, and analysis of variance (ANOVA). The data of post-treatment measurements will be analysed in agreement with the intention to treat principle. Assuming missing data will be missing at random, the Linear Mixed Modeling (LMM) procedure will be used for all analyses to estimate missing values. LMM includes incomplete cases in the analysis and employs restricted maximum likelihood estimation to calculate parameter estimates.

Effects between the experimental and control group on continuous measures will be calculated with Cohen's *d*. Cohen's *d *is computed by subtracting the mean post-test score of the control group from the average score of the experimental group and dividing the difference by the pooled standard deviation. Effect sizes of 0.8 can be assumed to be large, while effect sizes of 0.5 are moderate, and effect sizes of 0.2 are small [35]. Estimated data from the LMM procedure will be used to analyse effect sizes.

## Discussion

Internet based self-help for PD has proven to be effective. However, existing interventions focus on treating a full-blown disorder, while sub-clinical PD is highly prevalent and can develop into full-blown PD. This study is a randomised controlled trial aimed to evaluate an Internet based self-help intervention for sub-clinical and mild PD compared to a waiting list control group. The primary outcome measure is the severity of panic and anxiety symptoms. Secondary outcome measures include depression, quality of life, loss of production and health care consumption.

### Methodological considerations

The primary research question is whether DPO is an effective intervention for mild and sub-clinical PD. We hypothesise the experimental group will show more improvement on the outcome measures than the control group, because the experimental group receives an accessible intervention tailored for its specific mental health problems.

A limitation of this study is the low number of exclusion criteria. The presence of a comorbid disorder or substance abuse is not an exclusion criterion. Comorbidity may influence this study's outcome measures, because panic symptoms may not be the participant's primary mental health problem. However, in reality, many people who suffer from panic symptoms have comorbid mental health problems, especially other anxiety disorders and depression [6]. Therefore, it is preferable not to exclude participants based on DSM diagnoses for these disorders. An indication of the effects of comorbidity on the effectiveness of DPO could be obtained by mediator analyses.

Both a strength and a limitation of this study is that, apart from the diagnostic interview, the measurements are conducted online. Some evidence suggests psychometric properties may change when a test is conducted via the web [36,37]. Consequently, results of this study may differ from studies into panic interventions that applied paper-pencil questionnaires. On the other hand, the strength of online self-report measurements is the accessibility of participation [38]. Therefore, this study is feasible and should be able to answer the research questions.

## Conclusion

Existing online interventions for panic symptoms focus on treating full-blown PD, while sub-clinical PD is highly prevalent and may develop into a full-blown disorder. This study evaluates an Internet based self-help intervention for sub-clinical and mild PD. Results will contribute to the ongoing research into Internet based interventions and treatment of panic symptoms.

## Competing interests

The authors declare that they have no competing interests.

## Author's contributions

All authors have contributed to, read and approved this manuscript.
